# Synergistic metal–carbon interactions in Fe_3_O_4_/N-MWCNT composites for electro-Fenton processes

**DOI:** 10.1039/d5ra06118k

**Published:** 2025-11-25

**Authors:** Luis Alberto Romero-Orellana, Mercedes Teresita Oropeza-Guzmán, Luis Alberto Estudillo-Wong, Gabriel Alonso-Núñez, Hector Daniel Ibarra-Prieto, Adriana Jiménez-Vázquez, Yadira Gochi-Ponce

**Affiliations:** a Tecnológico Nacional de México/Instituto Tecnológico de Tijuana, Posgrado en Ciencias de la Ingeniería Blvd. Alberto Limón Padilla s/n, Mesa de Otay CP 22500 Tijuana B.C. Mexico; b Laboratorio de Electroquímica Ambiental y de Materiales, Departamento de Biociencias e Ingeniería, CIIEMAD, Instituto Politécnico Nacional Calle 30 de junio de 1520 s/n, Barrio la Laguna Ticomán Alcaldía GAM C.P. 07340 Ciudad de México Mexico; c Centro de Nanociencias y Nanotecnología, Universidad Nacional Autónoma de México (CNyN-UNAM) Km 107 Carretera Tijuana-Ensenada CP 22800 Ensenada B.C. Mexico; d Centro de Investigación en Materiales Avanzados, S.C. (CIMAV), Subsede Monterrey Av. Alianza Norte 202, Parque PIIT Apodaca 66628 Nuevo León Mexico; e División Académica de Ciencias Básicas, Universidad Juárez Autónoma de Tabasco Cunduacán Tabasco CP: 86690 Mexico

## Abstract

This work investigates the performance of graphitic nitrogen-doped multi-walled carbon nanotubes (N-MWCNT) decorated with Fe_3_O_4_ nanoparticles for the oxygen reduction reaction (ORR) and their application in the degradation of methyl orange (MO) using a heterogeneous electro-Fenton process. The combination of Fe_3_O_4_ and N-MWCNT enhances electrocatalytic activity through electronic metal-carbon interactions (EMCI), which promote charge transfer and improve electron mobility. Advanced characterization techniques, including TGA, TEM, XRD, Raman, XPS, UV-Vis, and electrochemical analysis, confirm the synergistic effects of combining graphitic N-MWCNT and Fe_3_O_4_ during a coprecipitation synthesis. DPR analysis reveals that the Fe_3_O_4_/N-MWCNT composites (MC1 and MC2) undergo a transition from semiconducting to metalloid behavior (thertherezation), supporting the improved electron transfer properties. Raman and XPS analyses further confirm the structural and electronic contributions of graphitic nitrogen in N-MWCNT and Fe_3_O_4_, reinforcing the composite's enhanced ORR efficiency. TEM and XRD analysis corroborated the anchorage of Fe_3_O_4_ in the composite, with crystallite particle sizes of 14.7 nm in MC1 and 16.8 nm in MC2. Electrochemical studies indicate that MC1 exhibits the highest electrochemically active surface area (25.1 cm^2^ mg_Fe_3_O_4__^−1^), mass activity (73.66 mA mg_Fe_3_O_4__^−1^), and turnover frequency (0.1768 s^−1^), indicating an increased number of active sites. Additionally, when composites are used as cathodic materials deposited by electrophoretic deposition (EPD), they effectively degrade 20 ppm of methyl orange at a neutral pH and a current density of 10 mA cm^−2^. MC1 achieved the highest degradation efficiency of 97.0% after 120 minutes in an electrode area of 12 cm^2^. This study provides new insights into how metal–carbon interactions at the nanoscale can be leveraged to engineer multifunctional catalysts for next-generation electrochemical systems.

## Abstract 2

Fe_3_O_4_/N-MWCNT enhances electron transfer through metal–carbon interactions, improving ORR activity and enabling efficient electro-Fenton degradation of methyl orange.

## Graphical abstract

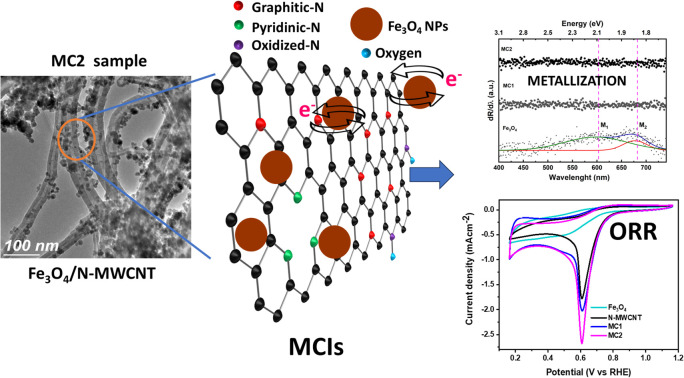

## Introduction

The development of efficient, optimized active sites and cost-effective electrocatalysts for ORR is still a challenge for advancing electrochemical processes related to both energy conversion and environmental applications.^1–5^ Analyzing the role of ORR in technologies such as fuel cells, metal-air batteries, and advanced oxidation processes for wastewater treatment reveals that oxygen reduction is often the rate-limiting step,^6–9^ thus the design of materials with active sites can lead to improving this fact. Platinum (Pt) catalysts have been the benchmark due to their structurally ordered activity.^10^ However, their high cost and scarcity have driven extensive research into non-precious metal alternatives.^11^

One of the most recent alternatives involves transition metal oxides supported on carbon-based materials, exhibiting high catalytic activity and environmental compatibility.^12,13^ Among these materials, magnetite (Fe_3_O_4_) has attracted considerable attention due to its low cost, magnetic properties, and ability to catalyze reactions under mild conditions.^14–16^ In addition, Fe_3_O_4_ possesses two Fe oxidation states (Fe^2+^/Fe^3+^) that create higher electrochemical potential sites, influencing the ORR mechanism. Nevertheless, the intrinsic ORR activity of Fe_3_O_4_ is limited compared to that of noble metals, motivating the exploration of composites that combine Fe_3_O_4_ with conductive carbon supports. This nanoscale strategy looks for a synergistic effect of electronic metal-carbon interactions (EMCI), thereby enhancing electron transfer and stability while preventing magnetite nanoparticle agglomeration.^17–20^ Moreover, recent studies have revealed that EMCI can induce structural alterations, such as metallization, whereby the electronic properties of carbon materials are modified by the presence of interfacial metal, thus changing the catalytic performance.^21,22^

Recently, new interfacial catalyst designs, such as boron-doped Co/Co_2_N, have demonstrated remarkable oxygen evolution reaction performance due to charge redistribution at heterointerfaces. *In situ* Raman studies of layered double hydroxides (LDH) highlight how interfacial dynamics directly influence catalytic pathways. These findings confirm that tuning interfacial charge transfer is a crucial strategy to optimize electrocatalytic activity.^23,24^

Carbon nanotubes (CNTs), particularly MWCNTs, are ideal candidates for catalytic support due to their superior properties, including high surface area, electrical conductivity, and robust structural stability.^25–27^ Incorporating Fe_3_O_4_ nanoparticles into MWCNTs establishes a conductive network, thus improving electron mobility and enhancing overall catalytic efficiency.^28–30^ Furthermore, MWCNTs doped with heteroatoms, such as nitrogen, substantially enhanced their catalytic potential by creating an electron-rich environment that facilitates the ORR.^30–33^

Alongside N-doping, other strategies based on biomass-derived carbons have shown significant promise. For instance, sugarcane bagasse-derived carbon supporting MoS_2_ nanosheets and lignin-assisted chestnut shell carbon/MoS_2_ composites improve electron transport and expose more catalytic sites, demonstrating the versatility of sustainable carbon supports for interfacial electrocatalysis.^34,35^

The combination of Fe_3_O_4_ and N-MWCNTs offers versatile, effective, and environmentally sustainable catalysts.^36,37^ Among various nanoscale doping strategies, incorporating graphitic nitrogen within the carbon lattice has been proven to enhance the ORR activity of CNT-based materials.^38,39^ MWCNTs-doped graphitic nitrogen enhances electrical conductivity by modifying their electronic structure.^40,41^ These structural changes also reinforce the interaction between Fe_3_O_4_ and N-doped MWCNTs, thus enhancing electron mobility and catalytic activity for the ORR.

Similarly, N-doped porous carbons encapsulating RuO_*x*_ nanoparticles have demonstrated pH-universal hydrogen evolution performance due to interfacial electron redistribution, further supporting the notion that heteroatom-doped carbon frameworks can modulate the electronic environment and stabilize metallic nanoparticles.^42^

While much research has examined the individual properties of Fe_3_O_4_ and nitrogen-doped carbon materials, fewer studies have explored the use of combined Fe_3_O_4_ and N-doped MWCNTs.^43,44^ Nevertheless, no one has prepared the Fe_3_O_4_/N-doped MWCNTs composite in a single step without using organic solvents, nor has an explanation been provided for EMCI. As an alternative approach to describe metal–carbon interaction and its influence in electronic charge transfer, some researchers have investigated the ultra-high temperature effect on metal support interactions in carbon-supported catalysts.^45^ However, the detailed influence of EMCI on ORR performance remains insufficiently understood.

Concerning the utilization of ORR in environmental applications, as heterogeneous electro-Fenton processes, Pormazar and Dalvand show an interesting proposal using activated carbon with magnetic nanoparticles packed in a stainless-steel basket as a cathode and a copper plate as an anode to degrade methyl orange. However, they do not consider the use of the catalyst metal–carbon interaction as a key factor in their results.^46^

This study investigates the effects of EMCI in N-doped MWCNTs enriched with graphitic nitrogen and decorated with Fe_3_O_4_ and their role in enhancing ORR performance. By utilizing advanced characterization techniques such as thermogravimetric analysis (TGA), transmission electron microscopy (TEM), X-ray photoelectron spectroscopy (XPS), Raman spectroscopy, UV-Vis diffuse reflectance spectroscopy (DRS), cyclic voltammetry (CV), and linear sweep voltammetry (LSV), we analyze the contributions of electronic structure and surface properties to the superior electrocatalytic activity of these nanocomposites. The environmental application and the effect of metal–carbon interaction of the N-doped MWCNTs enriched with graphitic nitrogen and decorated with Fe_3_O_4_ catalyst were done in a heterogeneous electro-Fenton process to degrade methyl orange.

### Experimental methods

#### Materials

All reagents used were of analytical grade purchased from Sigma-Aldrich, benzylamine 99.5%, ferrocene 98%, ferrous sulfate 99% (FeSO_4_·7H_2_O), ferric sulfate 79.2% (Fe_2_(SO_4_)_3_·*n*H_2_O), ammonium hydroxide 30% (NH_4_OH), *N*,*N*-dimethylformamide (DMF), Nafion (5 wt%), sodium sulfate (Na_2_SO_4_), methyl orange (MO) and deionized water with a specific conductivity of 2 × 10^−6^ S cm^−1^.

#### Synthesis of N-doped MWCNTs enriched with graphitic nitrogen decorated Fe_3_O_4_

The co-precipitation technique was used to synthesize N-doped and magnetite-decorated MWCNT. Initially, N-doped MWCNT (enriched with graphitic nitrogen) were synthesized by pyrolysis in a chemical vapor deposition reactor with 2.5 wt% ferrocene in 10 mL benzylamine with an argon flow of 0.5 L min^−1^ and 900 °C.^47^ Subsequently, the magnetic nanocomposite was prepared by suspending 100 mg of N-doped MWCNT in 20 mL of deionized water containing ferrous sulfate and ferric sulfate with two different molar ratios of iron salts: 0.15 : 0.30 and 0.50 : 1.00, labeled as MC1 and MC2, respectively. Calculating the weight proportion for each case (100 mg of N-doped MWCNT plus the used iron mass), MC1 must contain 26 wt% Fe_3_O_4_ and MC2 54 wt% Fe_3_O_4_.^14,48^ The reaction proceeded under a constant argon atmosphere with continuous stirring at 75 °C. Then, 1 mL of 8 M NH_4_OH aqueous solution was added to precipitate the magnetite for 30 min. Afterward, the precipitate was separated by magnetic decantation, washed three times with deionized water and ethanol, and left in an oven at 50 °C for 24 hours.

#### Raw and composite nanostructures characterization

Thermogravimetric analysis (TGA) was conducted in a TA instruments Q600-SDT with a heating ramp of 20 °C min^−1^ in O_2_. Transmission Electron Microscopy analysis was performed on a JEOL-2010 microscope. For structural analysis, Powder X-ray diffraction (XRD) patterns were taken on a Philips X'PERT MPD diffractometer using Cu-Kα radiation (*λ* = 1.54060 Å), in a range of 10–90° continuous scanning mode, with a step size of 0.05°, time per step of 30 s, and a count of 1400. Raman spectroscopy was conducted at ambient temperature using a confocal WITec alpha300 system with a 100× objective lens and a 300 lines per mm diffraction grating. The measurements were performed using a red laser excitation source (633 nm). X-ray photoelectron spectroscopy (XPS) was performed using a SPECS spectrometer with a PHOIBOS 150 WAL analyzer and an XR 50 Al kα X-ray monochromatic source. Samples were degassed at 10^−5^ mbar before transferring to the analysis chamber (residual pressure < 5 × 10^−9^ mbar). The binding energies were calibrated using the C 1s peak at 284.8 eV. After background subtraction and peak integration, surface composition and chemical states were analyzed using AAnalyzer (version 3.0) software. The diffuse reflectance spectrum (DRS) of the material was recorded on a Cary 5000 UV-Vis-NIR spectrophotometer, with the analysis region spanning 200–800 nm. Here, BaSO_4_ was used as a solvent to disperse the sample. The derivative peak fitting of diffuse reflectance (DPR) data was applied to analyze the spectra. The first derivative function applied to discrete data points is presented in eqn (1), where *R* represents the diffuse reflectance values and *λ* denotes the wavelength.^49,50^1
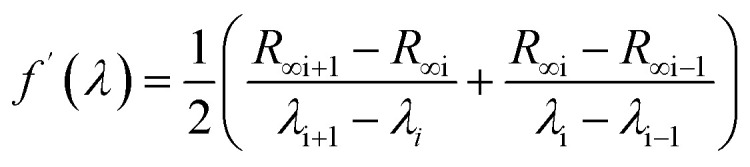


After processing numerical data, deconvolution of the signal was implemented by using a gauss function.

#### Electrochemical characterization

The electrochemical characterization was evaluated using a potentiostat (Autolab PGSTAT302N) and a rotating disk electrode (RDE). A three-electrode cell configuration was employed for these experiments: the working electrode was a glassy carbon electrode (geometric area of 0.07069 cm^2^), a platinum electrode served as the counter electrode, and an Ag/AgCl reference electrode (care was taken to rinse the electrode after each measurement). The ORR activity was assessed in an alkaline medium (pH = 13). Specifically, the catalyst material was deposited on the glassy carbon electrode by loading 4 µL of a previously prepared ink containing 5 mg of the catalyst in 1 mL of a solution composed of water and isopropyl alcohol, with a concentration of 0.1 wt% Nafion 117. Experimental analyses were conducted in a 0.1 M KOH solution, at the interval of potential region between −0.8 and 0.2 V *vs.* Ag/AgCl. All potentials were converted to the reversible hydrogen electrode (RHE) scale following the approach reported by Contreras *et al.*^47^ The kinetic current density and the number of electrons transferred during the oxygen reduction reaction (ORR) were determined using the Koutecky–Levich (K–L) model (eqn (2) and (3)).^51^2
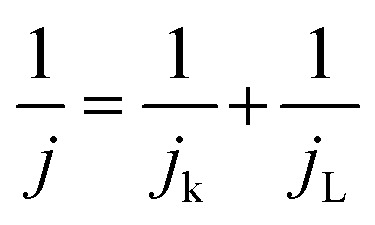

3 *j*_*L*_ = 0.062*nFAD*^2/3^*v*^−1/6^*ω*^1/2^CO_2_

here *j*, *j*_k_, and *j*_L_ represent the detected current, kinetic current, and limiting diffusion current density, respectively. In these equations, *n* denotes the number of electrons transferred, *F* is Faraday's constant (*F* = 96 485 C mol^−1^), *A* is the electrode area (*A* = 0.07069 cm^2^), *D* is the O_2_ diffusion coefficient (*D* = 1.9 × 10^−5^ cm^2^ s^−1^), *v* is kinematic viscosity of electrolyte (*v* = 1.13 × 10^−2^ cm^2^ s^−1^), *ω* is the rotating speed of the RDE, and CO_2_ is the bulk oxygen concentration (1.2 × 10^−3^ mol L^−1^).

### Fabrication of a heterogeneous electro Fenton cathode and methyl orange degradation

Electrophoretic deposition (EPD) was used to fabricate heterogeneous electro Fenton cathodes. A solvent mixture of 50 mg of catalyst was utilized with 95 mL of *N*,*N*-dimethylformamide (DMF), 5 mL of deionized water, and 50 µL of Nafion (5 wt%) under constant stirring. A Ti plate electrode (3 cm × 2 cm × 0.1 cm) was used as the substrate, with a platinum mesh of identical dimensions serving as the counter-electrode. A potential of 10 V was applied for 20 minutes.

Degradation experiments were conducted in 150 mL of an aqueous methyl orange solution (20 ppm) containing 50 mM Na_2_SO_4_ as the supporting electrolyte. A cathodic current of 10 mA cm^−2^ was applied for 120 min in a potentiostat/galvanostat Princeton Applied PARSTAT 2273. No pH adjustment was performed during the process. The degradation efficiency was monitored in a UV-visible spectrophotometer using 50 scans at 462 nm.

## Results and discussions

### Physicochemical and structural analysis

#### Thermogravimetric analysis

**Fig. 1:**
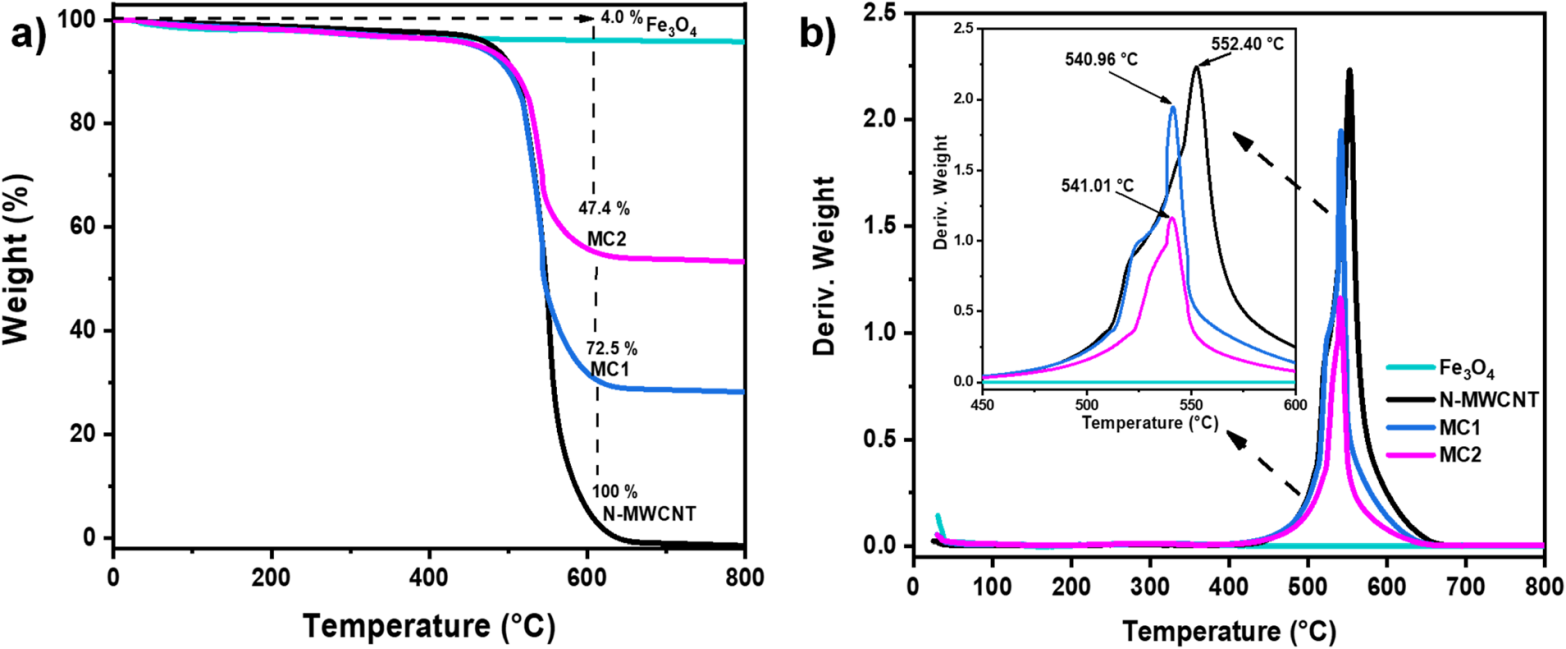
(a) TGA curve and (b) derivate weight of Fe_3_O_4_, N-MWCNT, MC1 and MC2.

The thermogravimetric profiles and their weight derivatives are presented in Fig. 1a and b for Fe_3_O_4_, N-MWCNT, MC1, and MC2 samples. First, a weight loss of approximately 4% is observed in the Fe_3_O_4_ sample, which could indicate stability over the analysis interval. In contrast, the N-MWCNTs begin to decompose at 552.40 °C, with total combustion of the sample occurring at 800 °C. For the MC1 and MC2 nanocomposites, these materials are approximately decomposed at approximately 541 °C, producing a weight loss of between 73% and 47%, respectively. The mass decrease during thermogravimetric analysis can be primarily attributed to the weakening of carbon–carbon bonds, leading to combustion and the formation of gaseous products from N-MWCNT.^52^ Moreover, these percentages closely correspond to those expected by mass balance calculations during composite preparation: 74% for MC1 and 46% for MC2 (see Experimental methods), indicating successful composite preparation. Besides, in the weight derivative plot, the temperature shift relative to the N-MWCNTs (*T* < 552 °C) is attributed to the presence of Fe_3_O_4_ nanoparticles, which play a crucial role by acting as an accelerator of the combustion process.^53^ TGA results are considered the initial approach to the EMCI, which will be further demonstrated with complementary physical characterizations.

#### Transmission electron microscopy

**Fig. 2:**
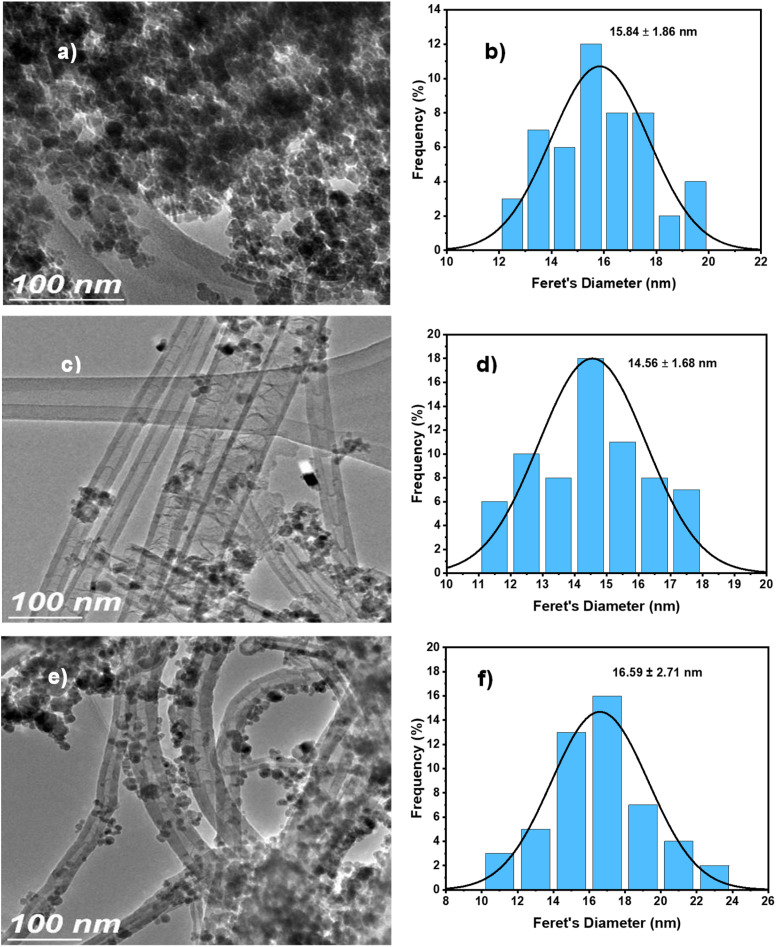
TEM micrographs and Feret diameter distribution of spherical (0D) morphology of (a and b) Fe_3_O_4_, (c and d) MC1, and (e and f) MC2.

Transmission electron microscopy was employed to analyze the morphology and particle size of raw materials and nanocomposites. TEM micrographs of Fe_3_O_4_, MC1, and MC2, as well as Feret diameter determination, are presented in Fig. 2. The analysis revealed that Fe_3_O_4_ exhibits a spherical morphology and denotes its presence according to the mass proportion in each case. For both composites (MC1 and MC2), the characteristic bamboo-like structure of nitrogen-doped carbon nanotubes is retained, with Fe_3_O_4_ nanoparticles ranging in size from 12 to 24 nm decorating the N-MWCNT.^32,54^ As shown in Fig. 2b, the distribution of Fe_3_O_4_ spherical morphology conforms to a normal distribution of diameter ratio, centered at 15.84 ± 1.86 nm. In contrast, the magnetite particle size on nitrogen-doped carbon nanotubes in the MC1 sample measures 14.56 ± 1.68 nm (Fig. 2d). Meanwhile, in the MC2 sample, it is 16.59 ± 2.71 (Fig. 2f). On the other hand, the average diameter of nitrogen-doped carbon nanotubes is approximately 40 nm. The difference in Fe_3_O_4_ nanoparticle size is likely related to the higher precursor concentration employed during the synthesis of MC2, which can influence nucleation and growth processes during co-precipitation. Similar behavior has been reported for magnetite nanoparticles, where increasing precursor concentration leads to slightly larger mean sizes and broader particle size distributions.^55^

**Fig. 3:**
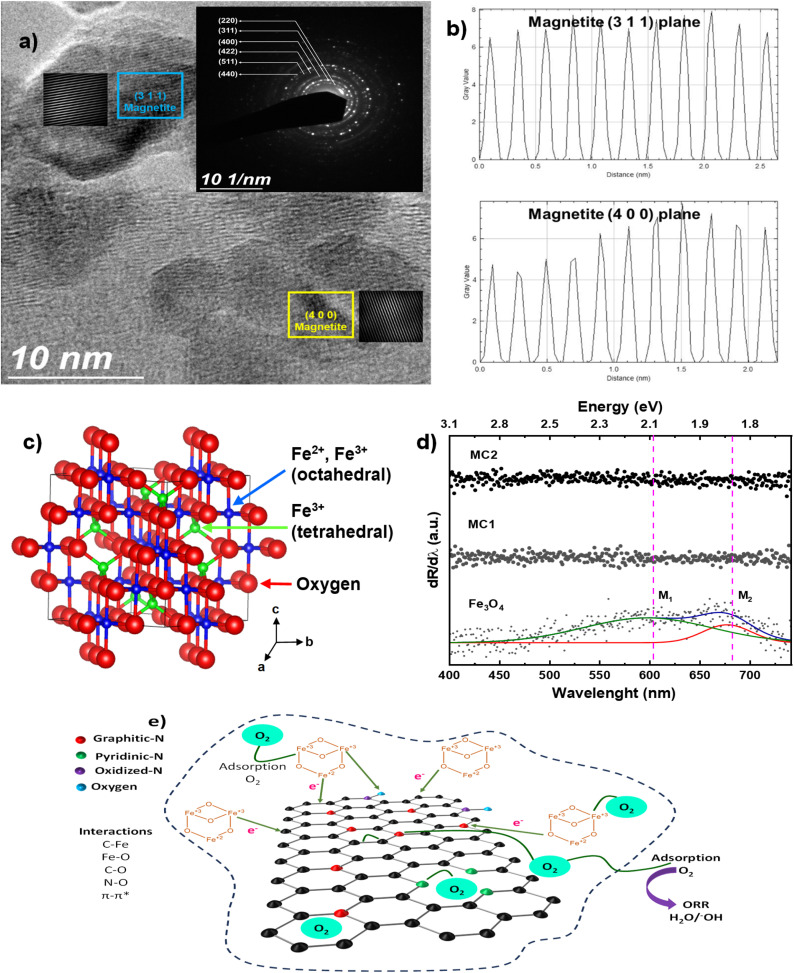
(a) TEM micrograph of the MC2 sample displaying magnetite crystalline planes (311) and (400), along with its SAED pattern shown in the inset, (b) grayscale profiles for magnetite crystalline planes (311) and (400), (c) schematic ball and line model for Fe_3_O_4_, and (d) metal transitions for Fe_3_O4, MC1 and MC2 samples. M1 and M2 peaks are related to transitions of Fe_3_O_4_. (e) Proposed schematic of Fe_3_O_4_/N-MWCNT. The electronic interaction facilitates charge mobility and oxygen reduction reaction *via* the four-electron pathway (4e^−^).

Additionally, the characterization of the MC2 sample by TEM and selected area electron diffraction (SAED) enabled the identification of the characteristic crystalline planes of the Fe_3_O_4_ anchored to the N-MWCNT. For instance, Fig. 3a shows the TEM micrograph, where regions with well-defined crystalline planes are observed, corresponding to the (311) and (400) planes of magnetite. These planes were further confirmed by inserting Inverse Fast Fourier Transform (IFFT) images, which highlight the atomic periodicity of these specific planes. The inset SAED pattern reveals well-defined Debye–Scherrer diffraction rings corresponding to the (220), (311), (400), (422), (511), and (440) planes.^16^ Furthermore, the periodicity of the (311) and (400) planes was validated through the gray intensity profiles obtained by IFFT, as presented in Fig. 3b. The periodic peaks observed in these plots correspond to the interplanar distance of the crystal lattice for magnetite. Thus, the findings indicate a cubic crystalline structure, as illustrated in the schematic ball-and-stick model (Fig. 3c), where octahedral Fe^2+^ and Fe^3+^ ions (blue), tetrahedral Fe^3+^ ions (green), and oxygen (red) are presented.^56^ TEM and SAED, applied to N-MWCNTs enriched with graphitic nitrogen, Fe_3_O_4_, and nanocomposites MC1 and MC2, confirm that magnetite crystals formed over the MWCNTs, resulting in localized metal-carbon interactions. However, the electronic importance of the catalytic nature of these new active sites needs to be demonstrated by physical and electrochemical characterizations.

UV-vis diffuse reflectance spectroscopy was employed to analyze the optical properties of magnetite (Fe_3_O_4_) and a composite formed with N-MWCNT. The spectra deconvolution was performed using the Fityk software, applying Gaussian functions for derivative peak fitting.^57^ This approach helped identify electronic transitions by fitting the derivative of the spectrum to resolve overlapping peaks. As shown in Fig. 3d, two signals, M1 and M2, were detected at 2.08 eV and 1.83 eV, respectively. These energy values indicate electronic transitions within the material, typically attributed to the charge transfer between Fe^2+^ and Fe^3+^ ions in the inverse spinel structure of magnetite. Precisely, 2.08 eV signal corresponds to a transition related to the charge transfer between octahedral Fe^2+^ and Fe^3+^ ions, while the 1.83 eV signal is associated with transitions between the conduction and valence bands as well as within Fe^3+^ ions.^58^ These signals are characteristic of a semiconducting behavior in magnetite and reflect its intrinsic electronic properties. However, no distinct signals were obtained during the deconvolution process when the analysis was extended to the carbon nanotubes and magnetite composite. The apparent disappearance of Fe_3_O_4_ absorption features in the composites (Fig. 3d) can be rationalized by two concurrent effects: (i) the strong broadband absorption of the N-MWCNT matrix, which elevates the baseline and masks Fe–O transitions, and (ii) interfacial charge transfer between Fe_3_O_4_ and the carbon framework, which alters the band structure and modifies optical transitions. Similar behavior has been reported for Fe_3_O_4_–GO composites, where spectral flattening and shifts arise from interfacial electronic coupling rather than a complete semiconductor-to-metal transition.^59,60^ Additionally, broad background absorption is intrinsic to carbon nanostructures such as CNTs and can surpass the intensity of oxide features. Therefore, this implies that the magnetite has transitioned from its typical semiconducting state to a more metallic conductive state when combined with the carbon nanotubes. This indicates that the iron oxide in the composite has undergone half-metallic.^61–63^ The highly conductive nature of MWCNTs is typically associated with this electron delocalization, effectively reducing or eliminating the semiconducting electronic transitions commonly observed in pure magnetite. Based on DFT calculations, magnetite is considered to have a half-metallic nature.^64^ As a result, the magnetite improves its electrical conductivity and behaves more like a metal, which explains the nanocomposite's lack of observable signals in the UV-vis spectrum. Instead of DFT calculations that can corroborate this hypothesis, Raman, XRD, XPS, and electrochemical analysis can also enlighten the EMCI effects observed in composites MC1 and MC2. Fig. 3e shows a schematic of a section of graphitic N-MWCNT with Fe_3_O_4_ nanoparticles inserted in the lattice. This diagram proposes a model based on TEM and UV-vis diffuse reflectance, suggesting the distribution of Fe_3_O_4_ along the carbon nanotube that physically promotes the hybridization of molecular bands of both materials, and impacting the electronic charge mobility on the newly created lattice. Later, the effects of surface electronic properties will be approached by more specific characterization techniques as Raman, XRD, XPS, and electrochemical analysis.

#### Raman spectroscopy analysis

**Fig. 4:**
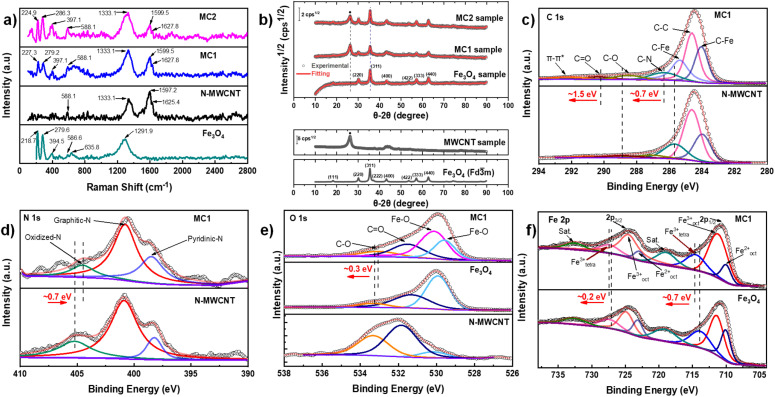
(a) Raman spectra of Fe_3_O_4_, N-MWCNT, MC1 and MC2, (b) pXRD and Rietveld Refinement Method of Fe_3_O_4_, MC1, and MC2 samples, showing fitted data in red color. MWCNT sample is used as support. The panel at the bottom depicts the simulated Fe_3_O_4_ phase. XPS spectra of (c) C 1s of N-MWCNT and MC1, (d) N 1s of N-MWCNT and MC1, (e) O 1s of N-MWCNT, Fe_3_O_4_ and MC1, and (f) Fe 2p of Fe_3_O_4_ and MC1.

Raman spectrometry is presented in Fig. 4a, which identifies the vibrational modes of Fe_3_O_4_, N-MWCNT, MC1, and MC2 samples. Raman spectrum of Fe_3_O_4_ nanoparticles specifically exhibited characteristic peaks at 218.7 cm^−1^ (T_2g_), 279.6 cm^−1^ (E_g_), 394.5 cm^−1^ (E_g_), 586.6 cm^−1^ (T_2g_), and 635.8 cm^−1^ (A_g1_), consistent with the inverse spinel structure of magnetite.^65–67^ These vibrational modes arise from Fe^2+^ and Fe^3+^ ions occupying octahedral and tetrahedral sites, confirming the crystalline nature of the nanoparticles, as corroborated by TEM analyses. Additionally, the D band was detected at 1291 cm^−1^, which is attributed to inherent heating effects due to laser irradiation.^68^

Although not directly related to the Fe_3_O_4_ structure, this signal highlights the presence of trace contaminants that are common during nanoparticle synthesis and handling. Moreover, the prominent T_2g_ mode at 586.6 cm^−1^, assigned to the symmetric stretching of Fe–O bonds, serves as a reference to track the structural integrity of Fe_3_O_4_ within the composites. In contrast, the Raman spectrum of the N-doped MWCNT exhibited the typical D band at 1333.1 cm^−1^, corresponding to disorder-induced vibrations from defects, and the G band at 1597.2 cm^−1^, representing the in-plane vibrations of sp^2^ hybridized carbon atoms.^69,70^ Furthermore, a nitrogen-induced D band at 1625.4 cm^−1^ was observed, providing confirmation of nitrogen doped into the carbon network. This band reflects nitrogen's incorporation into the carbon lattice, introducing electron-rich defects that enhance catalytic activity by providing additional active sites for the ORR. Notably, the *I*_D_/*I*_G_ ratio for the N-MWCNTs was 0.75, indicating moderate defect density, which balances catalytic activity without compromising the graphitic structure, thus contributing to the material's stability and performance.^71^

In the composites MC1 and MC2, the Raman spectra confirmed the retention of key vibrational modes from both Fe_3_O_4_ and N-MWCNTs, demonstrating the successful formation of the composite. The nitrogen-induced D band was also detected at 1627.8 cm^−1^ in both MC1 and MC2, confirming that the nitrogen-doping remained intact within the composite structure. Crucially, the *I*_D_/*I*_G_ ratio increases from 0.75 (N-MWCNT) to 1.06 (MC1) and 1.35 (MC2). To gauge the magnitude of disorder, the inter-defect distance *L*_D_ and defect density *n*_D_ was estimated using the Cançado (point-defect) relations for visible excitation and *L*_D_ ≥ 10 nm (eqn (4) and (5)):^72^4
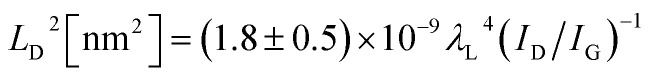
5
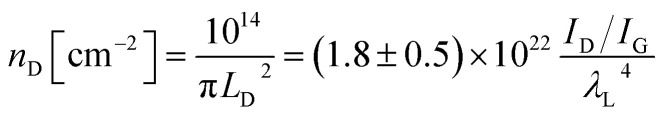
where *λ*_L_ = 633 nm. This yields *L*_D_ ≈ 16.5 nm and *n*_D_ ≈ 1.19 × 10^11^ cm^−2^ for MC1, and *L*_D_ ≈ 14.6 nm and *n*_D_ ≈ 1.51 × 10^11^ cm^−2^ for MC2. Nevertheless, under identical measurement conditions the comparative trend remains robust, demonstrating a higher defect density in MC2. This increase is consistent with denser Fe_3_O_4_ anchoring that generates edge/termination and local sp^3^ sites on CNT walls together with interfacial strain/charge transfer, as widely reported for Fe_3_O_4_/MWCNT hybrids where decoration increases *I*_D_/*I*_G_.^73,74^ These increases result from interfacial strain and localized disruptions caused by the interaction between the Fe_3_O_4_ nanoparticles and the carbon matrix, as observed in the TEM micrographs (well-dispersed Fe_3_O_4_ nanoparticles on the nanotube surfaces). It is expected that these additional defects will enhance catalytic activity by providing more oxygen adsorption sites and improving electron transfer pathways, thereby inducing superior ORR performance in the composite electrodes (see Surface Electrochemistry section). The T_2g_ mode at 588.1 cm^−1^ from Fe_3_O_4_ remained visible in both MC1 and MC2. However, with reduced intensity and slight broadening, the interfacial interaction (associated with charge transfer between the nanoparticles and the N-MWCNTs) is still detectable.

#### Powder X-ray diffraction analysis

Fig. 4b depicts the Powder X-ray Diffraction (PXRD) profiles from 10° to 90° for the N-MWCNTs, (Fe_3_O_4_), MC1, and MC2 samples. The N-MWCNT sample was doped with nitrogen and used as support. From Fig. 4b, two main peaks corresponding to the carbon and Fe_3_O_4_ phases can be observed. Specifically, for the Fe_3_O_4_ phase, eight planes were identified, which corresponds to Fe_3_O_4_(111), Fe_3_O_4_(220), Fe_3_O_4_(311), Fe_3_O_4_(222), Fe_3_O_4_(400), Fe_3_O_4_(422) and Fe_3_O_4_(511) planes. Additionally, the Rietveld Refinement Method was performed on Fe_3_O_4_, MC1, and MC2 samples to obtain the microstructural properties. The crystallography data was obtained from the Crystallography Open Database (COD) to ensure accuracy. The COD#900-6189 file was used for Fe_3_O_4_ (Magnetite, *Fd*3̄*m*).^75^ The fit (Gof) and *R*_wp_% parameters were 0.14 and 1.2% for all samples. These parameters confirmed the quality of the refinement and that no other phases were present. As summarized in Table 1, microstructural parameters, such as lattice parameters and crystallite average size, are considered. The micro-strain parameter was not considered because the value was close to zero. First, a value of 8.3554 ± 2 × 10^−3^ Å was calculated for the Fe_3_O_4_ sample for the lattice parameter. This value is lower than the MC1 and MC2 parameters. The observed increase in this value could be associated with a slight expansion in the lattice, which can be caused by magnetite anchorage onto the N-MWNCT. On the other hand, the value of the crystallite average size was found to range between 14 and 17 nm, as observed in the Feret's diameter, from TEM analysis. At this point, the crystallization of Fe_3_O_4_ on N-MWCNTs has been proven. However, the interfacial energy involved in the composite surface needs to be determined and related to an electrochemical reaction mechanism.

**Table 1:** Microstructural properties obtained by RRM for Fe_3_O_4_, MC1 and MC2 samples

Sample	Phases (%)	Lattice parameters Å	Crystallite average-size Nm	*R*_wp_%, Gof
Fe_3_O_4_	Fe_3_O_4_ (*Fd*3̄*m*)	8.3554 ± 2 × 10^−3^	15.8 ± 0.2	1.2, 0.14
MC1	Fe_3_O_4_ (*Fd*3̄*m*)	8.3591 ± 3 × 10^−3^	14.7 ± 0.2	1.2, 0.14
MC2	Fe_3_O_4_ (*Fd*3̄*m*)	8.3585 ± 2 × 10^−3^	16.8 ± 0.2	1.2, 0.14

#### XPS spectroscopy analysis

X-ray photoelectron spectroscopy was employed to determine the surface elemental composition and bonding configurations of Fe_3_O_4_, N-MWCNT, and the MC1 and MC2 composites. Fig. 4c–f and Fig. S1 display the XPS spectra for C 1s, N 1s, O 1s, and Fe 2p, respectively, for all materials. These analyses provide insights into the EMCI and how the incorporation of Fe_3_O_4_ influences the electronic structure of the N-MWCNT framework. The C 1s spectrum of N-MWCNT (Fig. 4c) exhibits characteristic peaks at 284.6 eV (C–C), 285.7 eV (C–N), 287.2 eV (C–O), 288.9 eV (C=O), and 291.1 eV (π–π*), confirming the presence of nitrogen functionalities and oxygen-containing groups.^54,76^ For MC1, the C 1s spectrum shows 284.0 eV (C–Fe), 284.6 eV (C–C), 285.4 eV (C–Fe), 286.4 eV (C–N), 288.5 eV (C–O), 290.4 eV (C=O), and 292.1 eV (π–π*). Compared to N-MWCNT, two C–Fe contributions (284.0 eV and 285.4 eV) indicate strong interactions between Fe_3_O_4_ and the carbon framework, confirming EMCI. The shift of C–N from 285.7 eV (N-MWCNT) to 286.4 eV (MC1) suggests modifications in nitrogen bonding states due to Fe_3_O_4_ incorporation. In MC2 (Fig. S1a), the spectrum presents an additional C–Fe peak at 285.2 eV, slightly shifted from MC1, indicating a different charge transfer dynamic between Fe_3_O_4_ and the N-MWCNT structure. The shifts in C–N (285.7 to 285.9 eV) and C=O (288.9 to 288.5 eV) suggest a higher degree of charge delocalization and interfacial stability than MC1.

The N 1s spectrum of N-MWCNT (Fig. 4d) shows peaks at 398.2 eV (pyridinic-N), 400.9 eV (graphitic-N), and 405.2 eV (oxidized-N), confirming the presence of nitrogen functional groups that enhance ORR catalytic activity.^77,78^ For MC1, the N 1s spectrum exhibits 398.5 eV (pyridinic-N), 400.8 eV (graphitic-N), and 404.5 eV (oxidized-N). Compared to N-MWCNT, the slight downshift in pyridinic-N suggests electron density redistribution caused by Fe_3_O_4_. The graphitic-N peak remains stable, indicating that nitrogen doping remains intact despite Fe_3_O_4_ decoration. For MC2 (Fig. S1b), the pyridinic-N peak shifts slightly (398.2 to 398.3 eV) while oxidized-N shifts to 404.9 eV, reinforcing the trend observed in MC1, but with a more pronounced electronic perturbation. These shifts suggest a stronger interfacial interaction in MC2, due to the higher Fe_3_O_4_ content (54 wt%) compared to MC1 (26 wt%).

Likewise, the O 1s spectrum of Fe_3_O_4_ (Fig. 4e) displays characteristic peaks at 529.9 eV (Fe–O), 531.4 eV (C=O), and 533.4 eV (C–O).^79,80^ For MC1, the spectrum reveals 529.6 eV (Fe–O), 530.2 eV (Fe–O), 531.5 eV (C=O), and 533.1 eV (C–O). The shift in the Fe–O peak from 529.9 eV (pure Fe_3_O_4_) to 529.6 eV (MC1) suggests partial electron transfer from Fe_3_O_4_ to the carbon matrix, reinforcing the presence of EMCI. In MC2 (Fig. S1c), Fe–O peaks appear at 529.7 and 530.4 eV, slightly shifted from MC1. The shift in C=O from 531.9 eV (N-MWCNT) to 531.8 eV (MC2) suggests that oxygen-containing groups are involved in charge redistribution, enhancing electrocatalytic activity.

Analyzing the Fe 2p spectrum of Fe_3_O_4_ in Fig. 4f, it exhibits characteristic peaks at 710.1 eV (Fe^2+^ octahedral), 711.5 eV (Fe^3+^ octahedral), 714.1 eV (Fe^3+^ tetrahedral), 719.4 eV (satellite), 723.2 eV (Fe^2+^ octahedral), 725.0 eV (Fe^3+^ octahedral), and 727.6 eV (Fe^3+^ tetrahedral), along with a satellite peak at 732.9 eV.^81–83^ For MC1, the Fe 2p spectrum reveals 710.1 eV (Fe^2+^ octahedral), 711.4 eV (Fe^3+^ octahedral), 714.8 eV (Fe^3+^ tetrahedral), 719.1 eV (satellite), 723.1 eV (Fe^2+^ octahedral), 724.9 eV (Fe^3+^ tetrahedral), and 727.5 eV (Fe^3+^ tetrahedral), with a satellite at 732.8 eV. Compared to Fe_3_O_4_, the Fe^3+^ tetrahedral peak shifts from 714.1 to 714.8 eV, suggesting enhanced electron transfer between Fe_3_O_4_ and N-MWCNTs through the reduction of Fe^3+^ to Fe^2+^ in MC1. In MC2 (Fig. S1d), the Fe^3+^ tetrahedral peak has a smoother shift from 714.1 to 714.4 eV, confirming a slightly weaker electron transfer effect than MC1. This may be attributed to particle agglomeration in MC2, resulting from its higher Fe_3_O_4_ mass, which diminishes direct interactions at the nanoscale between Fe_3_O_4_ and N-MWCNTs, as seen in TEM images (Fig. 2c and e).

The XPS confirms that both MC1 and MC2 exhibit EMCI, with Fe_3_O_4_ modifying the electronic structure of N-MWCNTs mainly due to the insertion of iron ions into the magnetite lattice. However, MC1 demonstrates a more pronounced electron transfer effect, as evidenced by a 0.7 eV positive shift in the Fe^3+^ tetrahedral peak compared to pristine Fe_3_O_4_, along with more prominent C–Fe bonding features, consistent with previously reported strong metal–carbon interactions that facilitate charge transfer and stabilize the metal centers.^17,21^ This suggests that MC1 may have better electroactivity due to a more homogeneous dispersion of Fe_3_O_4_ on the surface, compared to MC2. These findings also align with DRP analysis.

### Surface electrochemistry

**Fig. 5:**
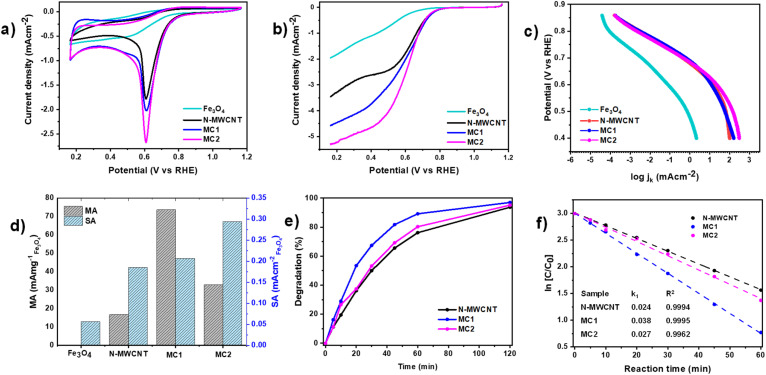
(a) CVs of Fe_3_O_4_, N-MWCNT, MC1, MC2 in O_2_-saturated 0.1 M KOH at 20 mV s^−1^, (b) LSVs of Fe_3_O_4_, N-MWCNT, MC1, MC2 in O_2_-saturated 0.1 M KOH at 1600 rpm and 5 mV s^−1^, (c) Tafel plots, (d) mass activity, specific activity for Fe_3_O_4_, N-MWCNT, MC1 and MC2. TGA profiles evaluated the mass of Fe_3_O_4_, (e) degradation of MO with initial concentration 20 ppm, 50 mM Na_2_SO_4_, *I*_c_ = 10 mA cm^−2^, neutral pH, and (f) Kinetic study of MO degradation by N-MWCNT, MC1, and MC2.

The electrochemical behavior of the synthesized composites was assessed through cyclic voltammetry and linear sweep voltammetry in an alkaline medium (0.1 M KOH) using an RDE setup. These analyses provide insights into the kinetics of the ORR and the associated electron transfer pathways involved for Fe_3_O_4_, N-MWCNT, and the composite materials MC1 and MC2. Fig. 5a presents the CV curves recorded at a scan rate of 20 mV s^−1^ raw and composite materials. The voltammograms exhibit distinct reduction peaks within the oxygen reduction region. Fe_3_O_4_ displays a reduction peak at 0.50 V with an onset potential of 0.64 V *vs.* RHE. In contrast, N-MWCNT, MC1, and MC2 composites demonstrated a shift towards a more positive reduction peak at 0.61 V with an onset potential at 0.70, 0.71, and 0.716 V *vs.* RHE for N-MWCNTs, MC1, and MC2, respectively. The onset potential was approached by the second derivative of the voltammogram, in a similar way as reported by Contreras *et al.*^47^ The observed shift in the reduction peak potential and the increased current density response point to an improvement of ORR. These changes are indeed related to the nanostructure (oxygen adsorption and electroactive sites), as well as the electronic charge transfer resulting from a synergistic interaction between Fe_3_O_4_ nanoparticles and the N-doped carbon. Considering that Fe_3_O_4_ nanoparticles are anchored along the N-MWCNTs, the structural modification creates new adsorption sites and assisted charge transfer sites due to the solid-state redox equilibrium of Fe^2+^/Fe^3+^ reached during cathodic polarization. This new approach to the ORR pathway may be suitable for the electro-Fenton process, where metallic species, such as Fe^2+^, can react with oxygen-adsorbed radicals to generate surface oxidative species, and recover Fe^2+^ sites through cathodic polarization. To demonstrate the electrocatalytic performance beyond what is expected from each component alone, *i.e.*, the electron transfer mechanism, LSV experiments were conducted at various rotation speeds (400–1600 rpm), and the corresponding Koutecky–Levich (K–L) plots were constructed. Fig. 5b illustrates the LSV curves for all electrocatalysts in an oxygen-saturated 0.1 M KOH solution at 1600 rpm and 5 mV s^−1^. The number of electrons transferred during the ORR was estimated based on the slopes of the K–L plots (Fig. S2). The results confirm that Fe_3_O_4_, MC1, and MC2 follow a complete 4-electron reduction pathway, consistent with the O_2_/OH^−^ redox mechanism. In contrast, N-MWCNT exhibits a lower electron transfer number of *ca.* 2.5, indicative of a partial reduction mechanism dominated by O_2_/HO_2_^−^.^47,84^ The Fe_3_O_4_ nanoparticles provide a redox-active interface, where Fe^2+^/Fe^3+^ transitions enable surface electron hopping, which couples with the conductive N-MWCNT network. This synergy facilitates interfacial electron delocalization and enhances charge transfer during ORR, thus promoting the 4e^−^ pathway observed in the Koutecky–Levich analysis.

In an alkaline medium, the selective adsorption of O_2_ by the Fe_3_O_4_ or carbon matrix dictates the initial reaction step. This begins with an initial 2-electron transfer, followed by synergistic interactions between Fe_3_O_4_ and the nitrogen-doped carbon, which promote the completion of the 4-electron pathway. This synergistic effect enhances electron transfer efficiency, leading to superior electrocatalytic activity.

The electrochemical active surface area (ECSA) was approached using the well-accepted cyclic voltammetry with a 10 mM K_3_Fe(CN)_6_ solution and 0.1 M KCl as electrolyte.^47^ Measurements were performed over a range of scan rates from 20 mV s^−1^ to 150 mV s^−1^ (Fig. S3). The electrochemical active area was calculated by applying the Randles–Sevcik eqn (6).^85^

6 *i*_p_ = 2.69 × 10^5^ × *n*^3/2^ × *A* × *D*^1/2^ × *v*^1/2^ × *C*

where *i*_p_ is the peak current (A), *n* is the number of electrons involved in the redox process, *A* is the electrochemical active area (cm^2^), *D* = 4.34 × 10–6 (cm^2^ s^−1^) is the diffusion coefficient, *v* denotes the scan rate, and *C* is the concentration (mol cm^−3^). Table 2 summarizes the ECSA, roughness (cm^2^_Fe_3_O_4__/cm^2^_geo_), wt% Fe_3_O_4_, and the turnover frequency (TOF). In the case of heterogeneous catalysis, it is recommended to report on the normalized surface area. In this case, it can be approached by dividing ECSA by the mass of Fe_3_O_4_ loading on the electrode. The composite MC1 exhibits the highest normalized ECSA at 25.1 cm^2^ mg^−1^, indicating that its structure and dispersion of Fe_3_O_4_ nanoparticles are optimized to expose a larger fraction of active sites relative to the catalyst mass.

**Table 2:** Electrochemical active surface area, roughness, wt% Fe_3_O_4_ and TOF

Sample	ECSA^a^		*Roughness	wt% Fe_3_O_4_		TOF
	cm^2^ mg_Fe_3_O_4__^−1^	cm_Fe_3_O_4__^2^		Nominal	TGA	s^−1^
Fe_3_O_4_^b^	0.2	0.004	17.67	100	—	0.0003
N-MWCNT^c^	6.4	0.128	1.810	—	—	0.0021
MC1^d^	25.1	0.138	1.952	26	27.5	0.1768
MC2^d^	7.9	0.083	1.174	54	52.6	0.0792

a Randles–Sevcik equation.

b Nominal value.

c Calculated by wt% N-MWCNT.

d TGA value, *calculated by cm_Fe_3_O_4__^2^/cm^2^_geo_.

Tafel plots, as shown in Fig. 5c, were constructed to assess the kinetic parameters of the ORR process. The extracted Tafel slopes show that potentials for all materials, at 0.7 V, were 65 mV dec^−1^ for Fe_3_O_4_, 54 mV dec^−1^ for N-MWCNT, 54 mV dec^−1^ for MC1, and 48 mV dec^−1^ for MC2. It is worth noting that the lower Tafel slope of MC2 reflects faster charge transfer kinetics at individual active sites, whereas its lower TOF compared to MC1 is related to its smaller electrochemical active surface area, indicating fewer effectively accessible sites due to Fe_3_O_4_ aggregation at higher loadings. These values indicate that the MC2 composite exhibits the most favorable charge transfer kinetics, attributed to the synergistic interaction between Fe_3_O_4_ and the graphitic nitrogen-doped MWCNTs, as well as the effect of the increased mass of magnetite, is detectable at lower electrode overpotentials. The observed reduction in Tafel slope values provides evidence for faster reaction kinetics. This is due to the enhanced accessibility of the active site and efficient charge transfer processes. The electrocatalytic performance of the materials was further analyzed by evaluating the specific activity (SA) and mass activity (MA), as shown in Fig. 5d. The SA, which represents the current per electrochemical surface area, was calculated using the eqn (7):^86^7
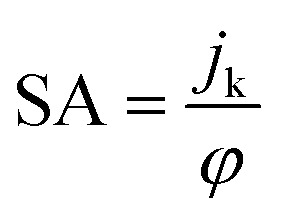
where *j*_k_ is the current density normalized by the geometric area and *φ* is the roughness, electrochemical surface area/geometric area ratio (cm_Fe_3_O_4__^2^/cm_geo_^2^).

The MA, which evaluates the activity per unit mass of the catalyst, was determined by eqn (8):^86^8
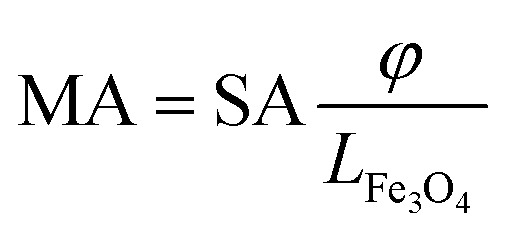
where *L*_Fe_3_O_4__ is the mass Fe_3_O_4_ loading of the catalyst per geometric surface area. The specific activity (SA) trend follows: MC2 > MC1 > N-MWCNT > Fe_3_O_4_, indicating that MC2 presents the highest intrinsic activity per electrochemically active surface area. In contrast, the mass activity (MA) trend follows MC1 > MC2 > N-MWCNT > Fe_3_O_4_, which correlates with the ECSA values (MC1 ≫ MC2), suggesting that the greater number of accessible active sites in MC1 compensates for its slightly lower intrinsic activity, resulting in superior overall mass activity.

The TOF, which quantifies the density of active sites in the catalyst, was determined using the eqn (9).^87^9
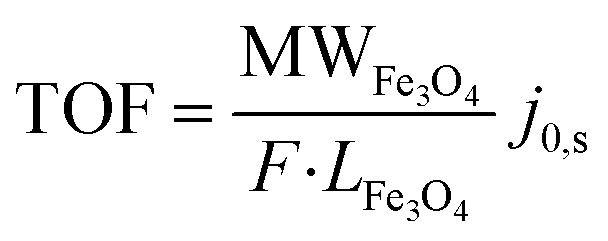
where *j*_0,s_ is the surface-specific exchange current density, *F* is the Faraday constant (96 485 C mol^−1^), MW_Fe_3_O_4__ and *L*_Fe_3_O_4__ represent the atomic mass and mass loading of Fe_3_O_4_. The TOF analysis confirms that MC1 and MC2 have a higher density of active sites (0.1768 and 0.0792 s^−1^, respectively) than Fe_3_O_4_ and N-MWCNT. The superior electrocatalytic performance is attributed to well-optimized structural and electronic properties, as verified by XPS, XRD, and TEM. Notably, the smaller Fe_3_O_4_ particle size in MC1 (14.7 nm) compared to MC2 (16.8 nm) contributes to its higher TOF, as reduced particle size increases the surface area-to-volume ratio, exposing a greater fraction of active electrocatalytic sites. Compared to previously reported Fe-based catalysts and Fe_3_O_4_ composites, our study demonstrates a significant improvement in TOF values, highlighting the enhanced catalytic efficiency of the MC1 and MC2 composites.^88,89^ These findings support the ORR mechanism. Fe-based catalysts follow an associative pathway involving O^2^/OH^−^intermediates. The EMCI in Fe_3_O_4_/N-MWCNT improves adsorption/desorption kinetics, facilitating efficient electron transfer. Incorporating Fe_3_O_4_ nanoparticles into graphitic N-doped MWCNT enhances electrocatalytic activity, making this composite a promising candidate for energy conversion and environmental applications.

### Methyl orange degradation using magnetic cathodes

The proof-of-concept study using magnetic nanocomposites as cathodes was performed for the heterogeneous electro-Fenton degradation of MO. Fig. 5e shows the degradation profile over 120 min for N-MWCNT, MC1, and MC2. The results demonstrate a strong influence of the catalyst composition on dye removal. MC1 achieved a degradation efficiency of 97.0%, followed by MC2 (95.15%) and N-MWCNT (93.7%). These trends correlate with their respective electrochemical surface areas, and Fe_3_O_4_ dispersion showed in the TEM studies. The degradation followed pseudo-first-order kinetics, as shown in Fig. 5f, with rate constants of 0.038 min^−1^ (MC1), 0.027 min^−1^ (MC2), and 0.024 min^−1^ (N-MWCNT), calculated from linear fits of ln(*C*/*C*_0_) *vs.* time. These values confirm that the synergistic EMCI in MC1 enhances ORR and promotes efficient heterogeneous electro-Fenton degradation of MO without requiring acidic conditions. From these experiments, it has been demonstrated that the integration of Fe_3_O_4_ and graphitic N-doped MWCNT (metal–carbon interaction) improves electron transfer, oxygen radical formation, and Fe^2+^/Fe^3+^ redox cycling, highlighting the key role of the material design in a specific application.

## Conclusions

N-MWCNT decorated with Fe_3_O_4_ nanoparticles were synthesized using a co-precipitation method with a nominal weight mass of MC1 (26 %wt) and MC2 (54 %wt). Based on physicochemical analysis, EMCI was confirmed during the synthesis process. First, TGA confirms the temperature shifting from 552.4 to 541 °C and the mass of magnetite contained in each composite. Besides, TEM-HR, XRD, Raman, and XPS analysis showed spherical morphology, an expansion of lattice parameter in Fe_3_O_4_, a higher defect density at the carbon framework, and a strong C–Fe interaction in the composite materials. Here, DPR analysis confirmed that those characteristics enhanced the electronic conductivity of the metal–carbon interaction within MC1 composite. In surface electrochemistry analysis, MC1 composite obtained an improvement in the electrochemical active surface area (25.1 cm^2^ mg_Fe_3_O_4__^−1^) and mass activity (73.66 mA mg_Fe_3_O_4__^−1^). Here, the turnover frequency (TOF) of the MC1 composite (0.1768 s^−1^) was 2.2239 times higher than that of the MC2 composite (0.0792 s^−1^). This trend is related to a better surface dispersion of Fe_3_O_4_, and lower crystallite-particle size (14.7 ± 0.2 nm) obtained in MC1 composite, which follows a four-electron reduction pathway. These properties and the electronic metal–carbon interaction make this material a strong candidate for large-scale environmental applications. In a heterogeneous electro-Fenton process, the MC1-based cathode effectively catalyzed the ORR to generate oxygen radicals while simultaneously serving as an iron source. This dual functionality enabled efficient degradation of organic pollutants such as methyl orange, achieving a removal efficiency of 97.0%. The synergistic interaction between the metal and carbon components plays a critical role in enhancing catalytic activity and broadening the material's application potential for wastewater treatment.

## Author contributions

Luis Alberto Romero-Orellana: methodology, investigation, writing – original draft. Luis Alberto Estudillo-Wong: methodology, software, writing. Gabriel Alonso-Núñez: resources, Hector Daniel Ibarra-Prieto: software, Adriana Jiménez-Vázquez: resources. Mercedes Teresita Oropeza-Guzmán: methodology, supervision. Yadira Gochi-Ponce: methodology, supervision.

## Conflicts of interest

There are no conflicts of interest to declare.

## Supplementary Material

RA-015-D5RA06118K-s001

## Data Availability

The data supporting this article have been included as part of the supplementary information (SI). Supplementary information: XPS spectra, the LSV curves at different rotation rates, and the CV voltammograms in 10 mM K_3_Fe(CN)_6_ for all materials. See DOI: https://doi.org/10.1039/d5ra06118k.
