# Supplementary material for: Synergistic metal–carbon interactions in Fe3O4/N-MWCNT composites for electro-Fenton processes
Source: RSC Adv. 2025 Nov 25;15(54):46367–79. doi: 10.1039/d5ra06118k (PMC12645587; doi:10.1039/d5ra06118k)
Supplement: RA-015-D5RA06118K-s001 [file RA-015-D5RA06118K-s001.pdf]

Supplementary Information (SI) for RSC Advances.
This journal is © The Royal Society of Chemistry 2025

Synergistic Metal–Carbon Interactions in Fe_3O_4/N-MWCNT Composites for Electro-Fenton Processes

Luis Alberto Romero-Orellana[a], Mercedes Teresita Oropeza-Guzmán[a], Luis Alberto Estudillo-Wong[b], Gabriel Alonso-Núñez[c], Héctor Daniel Ibarra-Prieto[d], Adriana Jiménez-Vázquez[e], Yadira Gochi-Ponce[a*].

[a]Tecnológico Nacional de México/Instituto Tecnológico de Tijuana, Posgrado en Ciencias de la Ingeniería. Blvd. Alberto Limon Padilla 18881, Cd Industrial, CP 22430, Tijuana, B.C., México.

[b]Laboratorio de Electroquímica Ambiental y de Materiales, Departamento de Biociencias e Ingeniería, CIIEMAD, Instituto Politécnico Nacional, Calle 30 de junio de 1520 s/n, Barrio la Laguna Ticomán Alcaldía GAM, C.P. 07340 Ciudad de México, México.

[c]Centro de Nanociencias y Nanotecnología, Universidad Nacional Autónoma de México (CNyN-UNAM), Km 107 Carretera Tijuana-Ensenada, CP 22800 Ensenada, B.C., México.

[d]Centro de Investigación en Materiales Avanzados, S.C. (CIMAV), Subsede Monterrey, Av. Alianza Norte 202, Parque PIIT, Apodaca 66628, Nuevo León, México

[e]División Académica de Ciencias Básicas, Universidad Juárez Autónoma de Tabasco, CP: 86690, Cunduacán, Tabasco, México

*Corresponding author: yadira.gochi@tectijuana.edu.mx

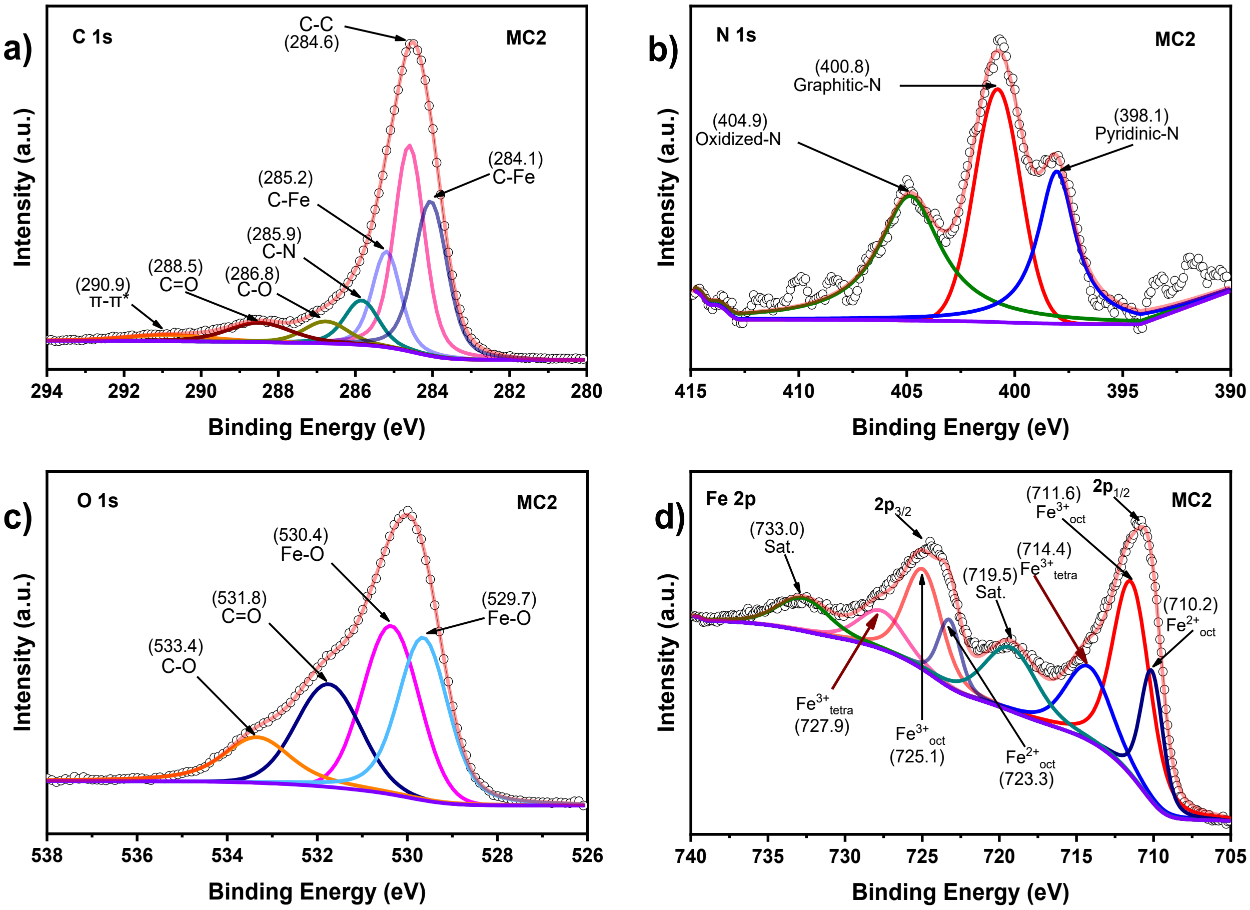

Figure 1S. XPS spectra of MC2 a) C 1s, b) N 1s. c) O 1s and d) Fe 2p.

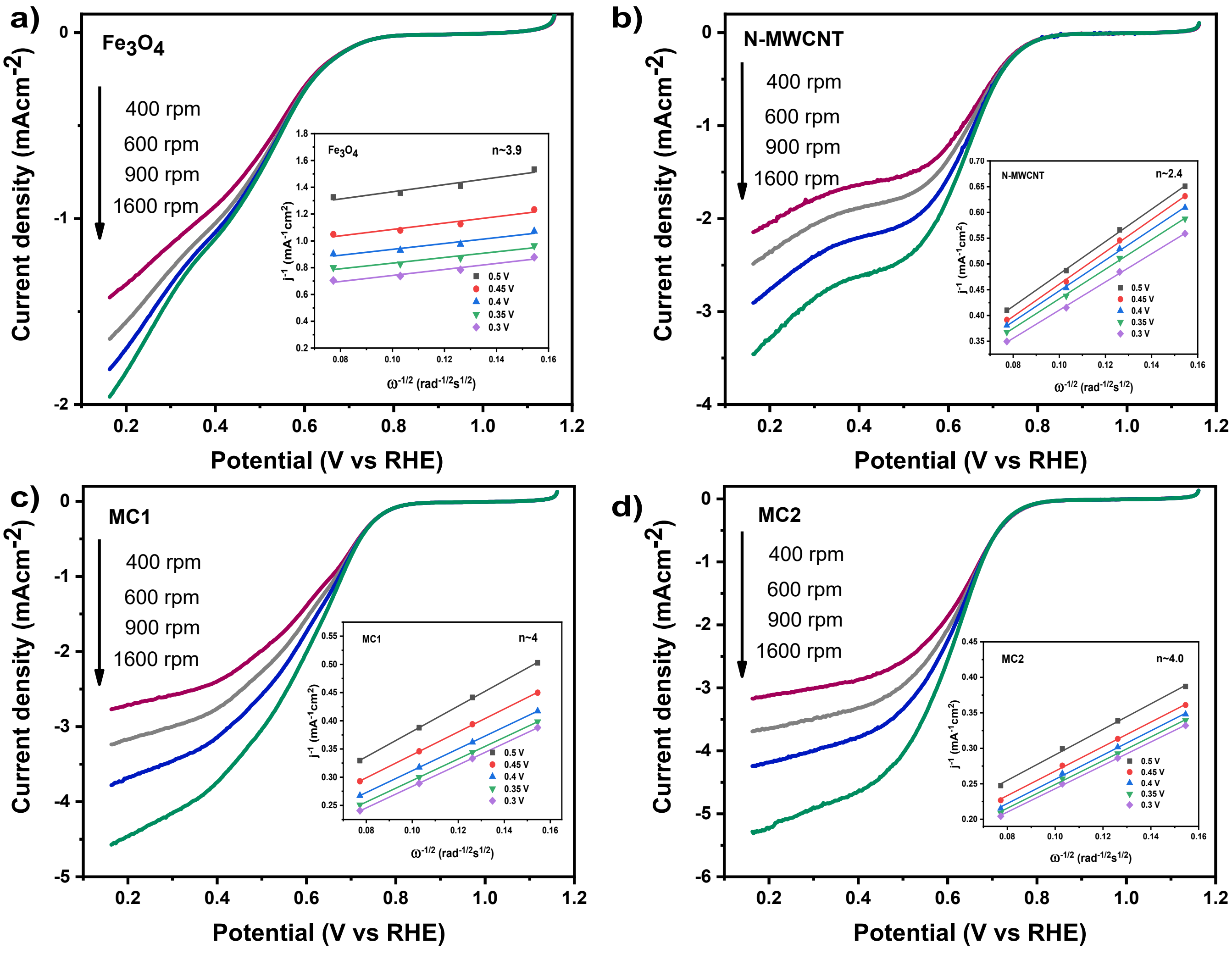

Figure 2S. (a) LSV at different rotation rates of Fe_3O_4 and inset corresponding plots at the potential range of 0.3 V to 0.5 V, (b) LSV at different rotation rates of N-MWCNT and inset corresponding plots at the potential range of 0.3 V to 0.5 V, (c) LSV at different rotation rates of MC1 and inset corresponding plots at the potential range of 0.3 V to 0.5 V, and (d) LSV at different rotation rates of MC2 and inset corresponding plots at the potential range of 0.3 V to 0.5 V.

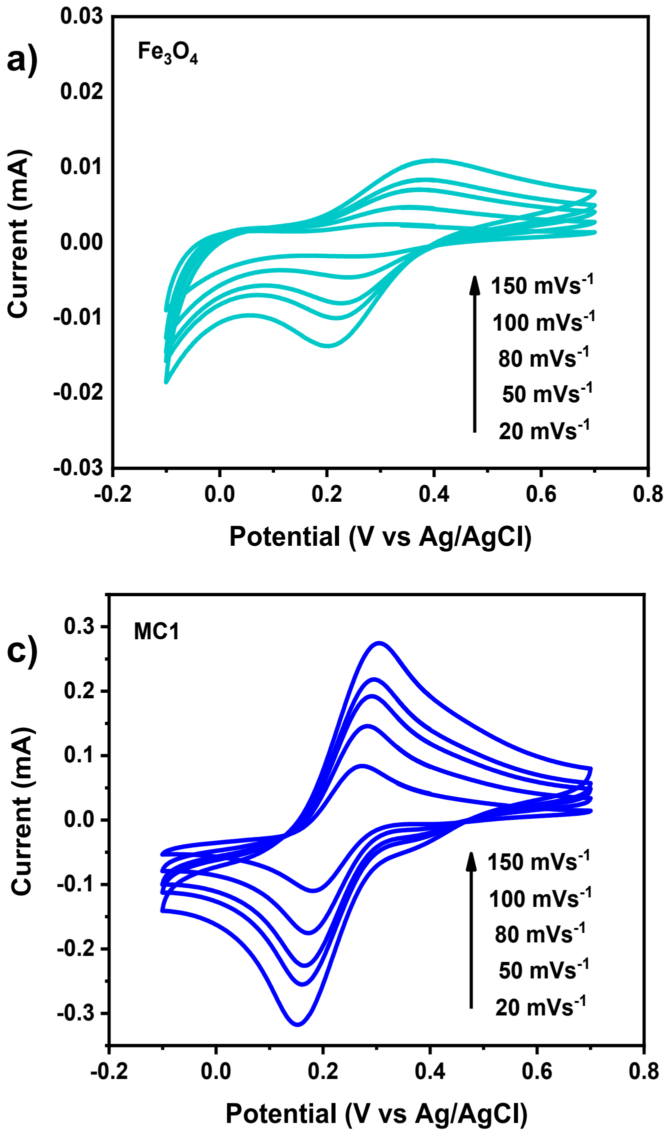

Figure 3S. a) CV voltammogram of Fe_3O_4 in 10 mM $K_3Fe(CN)_6$ solution and 0.1 M KCl as electrolyte from 20 mVs^{-1} to 150 mVs^{-1}, b) CV voltammogram of N-MWCNT in 10 mM $K_3Fe(CN)_6$ solution and 0.1 M KCl as electrolyte from 20 mVs^{-1} to 150 mVs^{-1}, c) CV voltammogram of MC1 in 10 mM $K_3Fe(CN)_6$ solution and 0.1 M KCl as electrolyte from 20 mVs^{-1} to 150 mVs^{-1}, d) CV voltammogram of MC2 in 10 mM $K_3Fe(CN)_6$ solution and 0.1 M KCl as electrolyte from 20 mVs^{-1} to 150 mVs^{-1}.
